# Prosthetic hernioplasty in elderly people: personal experience

**DOI:** 10.1186/1471-2482-13-S1-A22

**Published:** 2013-09-16

**Authors:** Fulvio Freda, Marco Scorzelli, Vincenzo Padovano, Domenico Larotonda, Raffaella Guerniero, Pasquale Petronella

**Affiliations:** 1 Second University of the Study of Naples, School of Medicine, Department of Gerontology, Geriatry and Metabolic Diseases, U.O. of Geriatric Surgery, Piazza Miraglia, 5 – 80138, Naples, Italy

## Background

Inguinal hernia is the most prevalent among the hernias that interested the abdominal wall with higher incidence in male than female, moreover it is more frequent in elderly than in younger patients. The incidence rises from 11 per 10,000 person-years aged 16-24 years to 200 per 10,000 person-years aged 65 years and above.

According to the literature the safest approach to geriatric patients is to perform hernioplasty in open surgery using local anaesthesia. The elderly patient has special features, typically related to the general conditions. Local anesthesia and postoperative analgesia are sufficient to solve the problems of old age. Over the last twenty years the surgical treatment of inguinal hernias has undergone significant changes. Today, the use of prothesis is the technique most used.

There are not many differences between the techniques *“tension free sutures less”* and *“tension free sutures no less”*.

## Materials

From January 2005 to December 2011 at the Department of Gerontology, Geriatrics and Metabolic Diseases at the Second University of Naples, we operated 293 patients for inguinal hernia, whose 92 elderly patients (older than 65 years) underwent surgical prosthetic hernioplasty for simple inguinal hernias. Patients with recurrence, bilateral hernia, large size hernia and all cases that underwent general anaesthesia were excluded from the study.

## Results

In the elderly there are multiple concomitant diseases compared with young, like respiratory or cardiovascular or metabolic diseases, for example.

We reported only 1 recurrence (1.08% of cases) with the *“tension free no suture less”* technique. As concerns postsurgical pain, no type of analgesic treatment was required for the most of cases treated.

Thanks to the prothesis and concomitant use of local anaesthetic you get a decrease of hospitalization time for the patients.

The duration of surgical treatment is about 45 min; the most of patients were discharged either one day after or two days after surgery due to severe concomitant pathologies such as ischemic cardiopathy, hypertension, renal insufficiency, obesity, hepatic cirrhosis, moderate-severe BPCO or decompensated diabetes mellitus.

The “*tension free suture less”* and *“tension free no suture less”* techniques also decreases morbidity connected to respiratory (atelectasis, infection) and circulatory complications (deep vein thrombosis, pulmonary embolism). If performed correctly, these techniques, which are based on reinforcing the posterior wall of the inguinal canal through the use of a prosthesis, display a recurrence rate of 0.1-0.3%.

The surgical prosthetic hernioplasty associated with the so-called *“nerve sparing”* technique - that is to identify and preserve the nerves during surgery - reduce postoperative pain and allow the patients to return soon to them normal physical activity.

## Conclusions

It is our opinion that the best treatment of inguinal hernia in the elderly patients is the surgical prosthetic hernioplasty associated with local anaesthesia. It is to be considered, moreover, that thanks to the use of “*nerve sparing”* technique you have a decrease in postoperative pain treatment.

Elective inguinal hernia repair under local anesthetic has a good outcome also in the elderly even if there are significant comorbidities. So in our opinion hernioplasty under local anesthesia is quite safe and feasible in patients over 65 years.
